# The Upcoming ^6^Li Isotope Requirements Might Be Supplied by a Microalgal Enrichment Process

**DOI:** 10.3390/microorganisms9081753

**Published:** 2021-08-17

**Authors:** Héctor M. Díaz-Alejo, Victoria López-Rodas, Camino García-Balboa, Francisco Tarín, Ana I. Barrado, Estefanía Conde, Eduardo Costas

**Affiliations:** 1 School of Veterinary Medicine, Complutense University of Madrid, Av. Puerta de Hierro s/n, 28040 Madrid, Spain; hectormd@ucm.es (H.M.D.-A.); vlrodas@ucm.es (V.L.-R.); camino@ucm.es (C.G.-B.); 2 ENUSA Advanced Industries S.A., S.M.E., Santiago Rusiñol 12, 28040 Madrid, Spain; ftg@enusa.es; 3 Spanish Research Centre for Energy, Environment and Technology (CIEMAT), Av. Complutense 40, 28040 Madrid, Spain; anaisabel.barrado@ciemat.es (A.I.B.); estefania.conde@ciemat.es (E.C.)

**Keywords:** lithium, fractionation, microalgae, biotechnology, stable isotopes

## Abstract

Lithium isotopes are essential for nuclear energy, but new enrichment methods are required. In this study, we considered biotechnology as a possibility. We assessed the Li fractionation capabilities of three Chlorophyte strains: *Chlamydomonas reinhardtii*, *Tetraselmis mediterranea*, and a freshwater Chlorophyte, *Desmodesmus* sp. These species were cultured in Li containing media and were analysed just after inoculation and after 3, 12, and 27 days. Li mass was determined using a Inductively Coupled Plasma Mass Spectrometer, and the isotope compositions were measured on a Thermo Element XR Inductively Coupled Plasma Mass Spectrometer. The maximum Li capture was observed at day 27 with *C. reinhardtii* (31.66 µg/g). *Desmodesmus* sp. reached the greatest Li fractionation, (δ^6^ = 85.4‰). All strains fractionated preferentially towards ^6^Li. More studies are required to find fitter species and to establish the optimal conditions for Li capture and fractionation. Nevertheless, this is the first step for a microalgal nuclear biotechnology.

## 1. Introduction

Future prospects for energy demand make it necessary to provide humans with an important ratio of KJ/(person × day) to satisfy the daily consumption all over the world. In 2019, energy consumption was over 160,000 TWh [1]. Additionally, the energy requirements must be supplied without posing a threat to human life and under environmentally sustainable criteria.

In that respect, fusion energy has become one of the most promising future sources of energy [2]. A project as relevant as ITER implies that researchers and engineers from different countries are approaching this challenge, aiming to obtain positive net energy from fusion reactions. To accomplish this, two reactants are required: two heavy hydrogen isotopes—deuterium, and tritium—as shown in Equation (1):

D + T → 4He + n + 17.6 MeV(1)

Deuterium may be obtained from seawater, where it is present at a concentration of 33 g/m^3^ [3]. Tritium is an isotope that is not abundant in nature but can be obtained from ^6^Li as in the process shown in Equation (2).

^6^Li + neutron → ^3^H (Tritium) + ^4^He (2)

Thus, lithium is a key element in the search for a green, efficient energy. From an isotopic point of view, lithium consists of two isotopes whose natural relative abundance is 7.59% for ^6^Li and 92.41% for ^7^Li [4]. Both lithium isotopes are strategic for the nuclear technology since ^7^Li is used to control the chemistry of the primary circuit in pressurized water nuclear reactors, while ^6^Li is used as neutron shield and detector in neutron beam facilities. Additionally, the advancement of nuclear fusion projects will create a high demand for this isotope to regenerate tritium. For this purpose, the enrichment needed in the ^6^Li isotope is around 30 to 90%, far above what is found naturally (7.59%) [5,6,7].

Since vast amounts of ^6^Li are required for fusion projects, the resultant ^7^Li is perfectly usable for battery production and nuclear fission reactors as an acidic control in pressurized water-moderated reactors (PWR) or as a coolant in GEN IV molten salt reactors since no tritium generation is desired in these systems [5]. The electric transportation sector is also interested in ^7^Li since it confers greater electromotive force, recharging speed, and useful life to batteries [8]. These synergistic demands for each of the lithium isotopes justify and reinforce the interest in developing efficient and scalable technologies of isotopic separation technologies [8].

To achieve fractionation, different methods based on physicochemical approaches have been developed, such as chemical, electrochemical, displacement chromatography, and laser-based methods [9]. Most of them have been proven at a laboratory scale and are very costly in terms of scaling and energy consumption. To our knowledge, however, Li fractionation has so far only been used industrially with the amalgam method [10], a chemical exchange process that uses high quantities of mercury that must be disposed of, posing an environmental and health risk [11,12].

Furthermore, nowadays, the majority of ^6^Li comes from a limited source and is produced in Oak Ridge [9], so envisioning the proximal future demand for ^6^Li, the implementation of plants to obtain huge amounts of enrichment must be achieved.

For these new plants to be more sustainable, green and more efficient technologies would be required [9]. To achieve this, nature could provide us with powerful knowledge since isotopes may be naturally fractionated during different biogeochemical processes.

Excluding hydrogen, the two isotopes of Li, ^6^Li, and ^7^Li, have the biggest relative mass difference (~16.6%) of any isotope pair, providing itself an advantage towards fractionation. Lithium fractionation in nature varies in geological and natural samples, with values for δ^7^ ([(R_measured_ − R_reference_)/(R_reference_)] × 1000) that range between around 30‰ in seawater to negative values in weathered soils and in soil solutions [13]. Taylor and Urey (1938) found an isotopic fractionation of 250‰ when Li-solutions percolate through a zeolite [14]. During weathering, fractionation occurs due to the primary dissolution of silicate rocks like granites and the formation of secondary minerals [15,16,17,18]. This can lead to producing large isotopic fractionation in terrestrial systems, with the variation of ^7^Li/^6^Li ranging from −20 to +40‰ [19,20] up to 80‰ [21].

Another possible fractionation mechanism is through biological systems. In mammals, including humans, Li isotopic fractionation process has been widely spotted [22,23,24]. The isotope relevance escalates to the point that they even exhibit different toxicity and lethality for ^6^Li and ^7^Li [25,26]. To show isotopic fractionation, a Li–organism interaction must exist. Many of the effects that Li can cause in living organisms may be explained by its competition with sodium, potassium, calcium, and magnesium due to its similar charge and size [27,28,29,30]. Because of its ubiquity in our environment, the ingestion of lithium in at least trace amounts is unavoidable [27]. Some plants have increased growth rates in the presence of lithium, but in other cases, lithium can also be toxic at high concentrations, so an optimal Li concentration for some organisms is likely to exist (as reviewed by Shahzad et al. [31]). Experiments conducted with bacteria such as *E. coli* or *Salmonella typhimurium* showed that Li^+^ ions as well as Na^+^ ions can be co-transported with trimethylglycine [32]. Additionally, Li^+^ could have a stimulating effect on proline transport in E. coli [33]. As such, the relationship between lithium and living organisms is, even if not essential, existent.

Among the living beings that can potentially be used in biotechnological processes, microorganisms have a predominant role. The fractionation ability of microorganisms has been widely demonstrated [34,35,36,37,38,39,40]. Lithium, albeit not widely studied, is no exception, as one study testing different bacteria found Li isotopic fractionation with different rates between species [41]. However, to our knowledge, no further studies on microbiological fractionation were found to be related to this element.

Microalgae have great acceptance and usefulness for biotechnological applications due to their properties: their ability to adapt to extreme environments [42,43,44,45,46,47,48,49], their ubiquity, their relatively easy culture conditions, and their ability capture CO_2_ while growing, helping to alleviate ongoing climate change (reviewed by Kumar et al. [50]). Additionally, related to our subject, they are well suited for metal uptake processes, either as dead biomass or while using them alive [51,52,53,54,55,56,57]. Previous studies allowed us to prove the fractionation of ^235^U and ^238^U employing a microalga isolated from a polluted extreme uranium mining environment with a maximum δ^235^ of 540‰ [58]. Additionally, some studies revealed the possible use of microalgae to recover lithium from a solution [54,59], but no further studies deepening this field have been found. Considering the valuable and promising properties of microalgae in terms of biotechnology, this study aimed to determine whether these microorganisms can fractionate lithium isotopes in laboratory conditions.

## 2. Materials and Methods

### 2.1. Microalgae Strains and Growth Conditions

A total of three Chlorophyta microalgae strains were used, two freshwater wildtype strains, an unidentified Chlorophyta (CAFE strain), and *Chlamydomonas reinhardtii* Dangerad (ChlA strain) and one marine wildtype strain, *Tetraselmis mediterranea* (Lucksch) R.E. Norris, Hori & Chihara (strain TmS1), was also used. The three strains were obtained from the Universidad Complutense de Madrid microalgae culture collection. The CAFE strain was isolated from the Entreuka Guelta (SE Mauritania); the ChlA strain was isolated from Doñana National Park (SW, Spain), and the TmS1 strain was isolated from waters off the eastern coast of Sardinia (Italy). The ChlA and TmS1 strains were selected as they belong to Chlorophyte genera used as model systems for freshwater and marine eukaryotic microalgae [60,61]. The CAFE strain was identified in this study, described in CAFE strain identification as *Desmodesmus* sp. and was chosen as a random strain to increase basic knowledge in the field.

Under laboratory conditions, strains were grown in 100 mL cell culture flasks (Greiner; Bio-One Inc., Longwood, NJ, USA) with 20 mL of BG-11 medium (Sigma-Aldrich Chemie, Taufkirchen, Germany) for the freshwater algae, and filtered seawater enriched with Guillard’s F/2 broth (Sigma-Aldrich Chemie, Taufkirchen, Germany) was used for the TmS1 strain. Culture media was prepared according to the manufacturers’ instructions. To keep a mid-log-exponential growth, the strains were transferred to a new culture flask within 20 days of its prior transfer. Cultures were maintained at 22 °C with continuous illumination at 80 µm/m^2^·s^1^ within the wavelength of 400–700 nm. For the experiments, LiCl (Sigma-Aldrich, BioXtra, ≥99.0% purity) was dissolved in the freshwater and marine media.

### 2.2. CAFE Strain Identification

An isolated CAFE culture was sent to Secugen S.L. (Madrid, Spain) for DNA extraction, amplification of 18S RNA and ITS genes, and sequencing from both ends. These genes have been successfully used in previous Chlorophyte identifications.

The DNA for the PCR was extracted by centrifuging 100 µL of liquid culture and inserting the pellet in an FTA^®^ Card. Forward and reverse gene sequences were amplified using the primers indicated in Table 1.

The 18S gene PCR was conducted under the following conditions: 95 °C 15 min + (95 °C 30 s + 55 °C 40 s + 72 °C 2 min) × 35 + 72 °C 10 min. For the ITS region, the following protocol was applied: 95 °C 15 min + (95 °C 30 s + 53 °C 40 s + 72 °C 1 min) × 35 + 72 °C 10 min. For both protocols, the TaqGold (Applied Biosystems™, Thermo Fisher Scientific Inc., Waltham, MA, USA) polymerase was used. PCR products were checked by visualization in agarose gel. DNA sequencing from both ends was conducted using the reactant BigDye 3.1 followed by capillary electrophoresis in an automatic sequencer ABI 3730xl. The obtained sequences were assembled and edited using the BioEdit software. A consensus sequence conducted for 18S and the ITS genes was compared using the Basic Local Alignment Search Tool (BLAST), a National Center for Biotechnology Information (NCBI) database.

The final strain identification was based on the 18S ribosomal RNA gene, internal transcribed spacer (ITS), and by morphological comparison with the AlgaeBase (www.algaebase.org) (accessed date: 10 September 2020) [64,65,66]. Internal transcribed spacer (ITS) and 18S ribosomal RNA genes obtained from the CAFE isolate were assembled, edited, and deposited in GenBank.

### 2.3. Lithium Uptake and Isotope Fractionation Trials

The lithium uptake and Li-isotope fractionation behaviour of the three Chlorophytes was conducted in a bioassay. Cultures were prepared in flasks containing 20 mL of the respective culture media. Lithium reached a concentration of 4.2 mg/L in BG-11 culture medium and a concentration of 2.5 mg/L in F2 medium. Additionally, one control without cells for each medium was established. The initial cellular inoculum was of approximately 8 × 10^5^ cells for TmS1 and CAFE and approximately 7 × 10^5^ cells for ChlA. Cell densities were estimated by counting with counting chambers (Fast Read; Biosigma, Venice, Italy) under a microscope Zeiss 47 30 12-9902 (Zeiss, Oberkochen, Germany).

The bioassay was conducted in aerobic conditions at 22 °C and pH 8.11 for F/2 and at 5.82 for BG/11 cultures. A total of four cultures were prepared for each strain, and each culture was to be centrifuged at a specific time: immediately after inoculation (considered 0 days) or after 3, 12, or 27 days. The whole volume of each culture was centrifuged at 4000 rpm for 15 min. The pellets were frozen until analysis. Solutions of BG-11 and F/2 enriched with lithium were also analysed during the experimental times to serve as chemical control. Isotopic analysis and total Li quantifications were performed at CIEMAT (Spanish Research Centre for Energy, Environment, and Technology). Li isotopic relationship results were obtained by averaging the data obtained from two pseudo-replicate analyses of each sample.

### 2.4. Analytical Procedure

#### 2.4.1. Sample Dissolution and Lithium Separation

Algal pellets (mg) were subjected to acid digestion with 5 mL of a HNO_3_-H_2_O_2_ mixture (4:1 *v*/*v*) in Teflon beakers and gentle heating on a hotplate until a transparent solution was achieved. Sample solutions were then evaporated until almost dry, and the residues were dissolved in 3 M HNO_3_. One millilitre of this solution was brought to a final volume of 10 mL with MilliQ water to quantify the Li by quadrupole-based ICP-MS (Q-ICP-MS) using the external calibration and internal standard method. The drying operation was repeated, with the remaining volume and the residue being dissolved in 2 mL of HCl 0.2 M, which was used for lithium isotopic analysis after lithium purification through chromatography.

#### 2.4.2. Chromatography

The separation of lithium from other matrices is crucial for precise Li isotope measurement. This step was conducted in a single-step chromatographic separation using 1.5 cm polypropylene columns (Bio-Rad Laboratories, Inc., Hercules, CA, USA) packed with cation-exchange resin, Dowex 50W-X8 (50–100 mesh size).

Prior to sample loading, the columns were pre-washed with 20 mL of 6 M HCl and were then conditioned with 10 mL of 0.2 M HCl. Samples in 0.2 M HCl medium were loaded and were subsequently eluted with 40 mL of 0.2 M HCl, with the eluate volume ranging from 20 mL to 32 mL of the corresponding to lithium fraction. These 12 mL were collected and were gently evaporated until dry, after which the residue was dissolved in 5 mL of 2% HNO_3_ (*v*/*v*). 

The lithium solution of each sample was quantified, and its concentration was adjusted by dilution in 2% HNO_3_ to an approximate concentration of 1 ppb before isotopic mass spectrometry analysis.

#### 2.4.3. Mass Spectrometry

The lithium concentration in samples was determined by Inductively Coupled Plasma Mass Spectrometry (ICP-MS) using a quadrupole instrument equipped with a collision cell (iCAP Q, Thermo Fisher Scientific Inc., Waltham, MA, USA) and by applying the external calibration quantification method and internal standardization. The standard calibration solutions were prepared daily by dilution from a 1000 mg/L Li stock solution. According to the concentration obtained in each sample, these were diluted to a Li content of 1–2 ng/mL.

Isotope compositions were measured on an Element 2 ICP-MS (Thermo Fisher Scientific). Tuning parameters are adjusted before the analyses to maximize instrument sensitivity and stability.

The ^6^Li/^7^Li ratio was measured using a sample-standard bracketing method, where a blank and a certified isotopic standard (IRMM-016, Joint Research Centre European Commission) were measured before and after each sample to correct for the instrumental drift and mass bias. The isotope standard was prepared by matching its concentration with that of the sample, that is, 1 µg/L, because an uneven concentration between the sample and the bracketing standards affects the accuracy of the Li isotope analysis. Additionally, blanks were systematically determined and were negligible. The uncertainty associated with the measurement of all ^6^Li/^7^Li isotopic ratios was less than their variation between samples.

## 3. Results

### 3.1. CAFE Strain Identification

The CAFE strain was identified as belonging to the genus Desmodesmus. The identification was based both on the ITS and 18S RNA gene sequences, and on morphological comparison with the AlgaeBase (algaebase.org) (accessed date: 10 September 2020).

Under the BLAST search, both the Desmodesmus and Acutodesmus genera showed an identity over 99% with the sequenced regions. The final classification of CAFE as Desmodesmus and not Acutodesmus was determined by morphological comparison with the AlgaeBase. Species-level identification was not reached. The obtained sequences were uploaded to GenBank with the accession numbers MT462646 for the ITS region and with the number MW185780 for the small subunit ribosomal RNA gene.

### 3.2. Lithium Uptake

Lithium concentrations in the culture media were analysed, showing 4.2 mg/L of Li in BG-11 + Li and 2.5 mg/L in F2 + Li.

The measure of the Li concentration in the assay confirms that the three microalgae that were tested were apt for lithium uptake (Table 2).

### 3.3. Isotope Fractionation Trials

The enrichment factor δ^6^ was calculated according to Equation (3), considering R_reference_ as the isotopic ratio measured in each specific control media. The calculated enrichment factor for each alga is detailed in Table 3.

δ = [(R_measured_ − R_reference_)/(R_reference_)] × 1000 (3)

## 4. Discussion

### 4.1. Lithium Capture

The three strains captured the most in the culture at day 27, but huge differences were found among strains and, arguably, the maximum Li capture was not reached during the period in question. ChlA showed the greatest capture followed by CAFE. According to Li mass relative to wet biomass, TmS1 and CAFE showed a similar uptake without great differences between them. ChlA again proved to be the most effective in incorporating lithium from solution, with a sharp change from day 12 to 27. For this strain, even if no great biomass increase occurred between days 3 and 27, the total Li mass incremented almost eight-fold. According to our results, the optimal moment for biomass recovery would be at day 27. However, further studies need to be conducted to determine on which day the maximum uptake is achieved.

Previous studies conducted with Chlorophytes demonstrated that microalgae can be a potent tool for Li removal from Li polluted waters [59] or from wastewater where Li and Rb are both present [54]. El Naggar et al. [59] evaluated the conditions that yielded the maximum Li capture using the Chlorophyte *Oocystis solitaria*, considering pH, lithium concentration, and temperature the most influential factors. A maximum of 99.95% Li removal was achieved in that assay. Presumably, the maximal uptake conditions would highly depend on the microorganisms used, as each strain has its peculiarities. In our assay, the experimental environment was the same as the conditions in which the culture collection was maintained, thus not causing stress.

**Figure 1:**
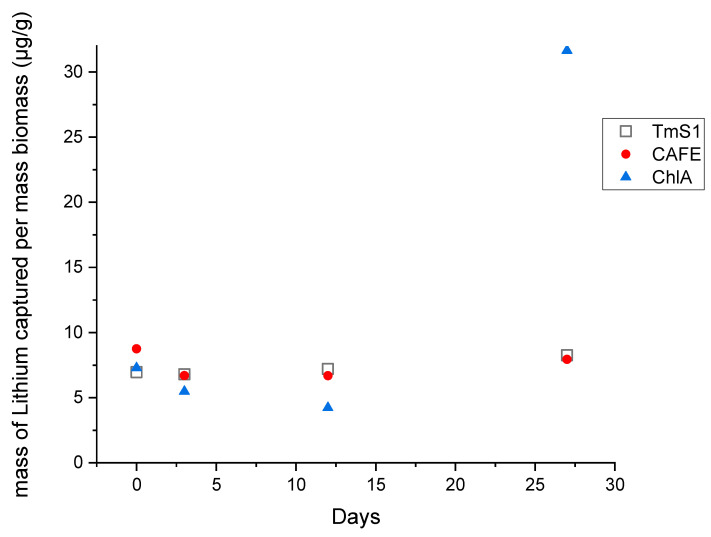
Li capture relative to total biomass in the experimental cultures with additional Li.

Lithium uptake was observed in the three strains within minutes from inoculation, but differences existed. On days 3 and 12, a slight Li-content decline was detected in the CAFE strain, while ChlA depletion was stronger. TmS1 did not show evidence of Li loss. During the last days of the trial, a slight increase in Li capture was observed in CAFE, but it was not enough to restore the initial levels (Figure 1). ChlA took up a total of 6.098 µg, which represents 31.66 ng/g of lithium in wet biomass. That amounts to around four times more lithium than the maximum taken up in the case of CAFE or TmS1. 

According to Jakobsson et al. [27], lithium uptake may be explained by its competition with sodium, using the same entrance mechanisms; most of its biological effects can be attributed to competition with Na, Mg, and K sites. Na and Li ions have similar properties such as small size, the same acid-base behaviour, and the same number of electrons in the last electron shell [67], which accounts for their similar behaviour in biological systems. High salt concentrations may result in being toxic to microalgae, so the species thriving in saline environments may keep sodium ions away from the cells relying on active transport or may conceal the ions in vacuoles far from metabolic processes as well [68]. Li^+^ ions in cells could follow a similar path. A recent study also showed that in Chlorella vulgaris, the K^+^ concentration affected Li uptake [54]. In this latter study, Li biosorption was observed followed by desorption in the following days as well as a trend where a decrease in the K^+^ medium concentration increased the Li uptake in a media where Rb^+^ was also present. The total Li uptake when K^+^ concentration was reduced to 60% was 4.70 mg/g dry mass, a 4.48-fold increase compared to the medium with non-altered K^+^ concentration. This observation, if further studies are conducted, could lead towards the design of culture media for biotechnological Li capture purposes. Furthermore, this observation extends the idea of the Li behaviour in microbial systems as an incidental substitute for biological ions, such as K^+^, in microalgae.

### 4.2. Fractionation

Since biological isotopic fractionation is a relatively frequent natural process [40], we aimed to obtain lithium isotopic fractionation by taking advantage of microalgae properties. Lithium isotopic fractionation based on the activity of bacteria was already published by Sakaguchi and Tomita (2000) [41], where a maximum δ^6^ of 58.7‰ was obtained using the bacteria *Bacillus megaterium*. However, from that point on, little effort and resources have been dedicated to advancing the field of microbial lithium fractionation, as we have not encountered any other publications respecting this topic, particularly in terms of microalgal lithium fractionation. Furthermore, until recently, neither the growth of plants [69] nor phytoplankton [70] had shown a significant Li fractionation ability in naturally occurring lithium concentrations. Nevertheless, a recent study revealed that plants are indeed capable of fractioning ^6^Li with, e.g., an observed δ^7^ of 12.2‰ in rhizomes from a humid region, whereas the δ^7^ observed in the grass blades was 6.2‰ [71]. In our study we extended this knowledge, confirming that, under laboratory conditions, some microalgae, or, more precisely, our three strains belonging to the phylum Chlorophyta, can differentiate between lithium isotopes. On the contrary, one study conducted in the Laxa river in Iceland pinpointed that microalgae did not affect Li fractionation in this natural environment [70]. Many different variables could affect the different observed outcomes such as natural and laboratory conditions, Li concentration, algae species, biomass, or geochemical processes that may be involved in natural environments. 

**Figure 2:**
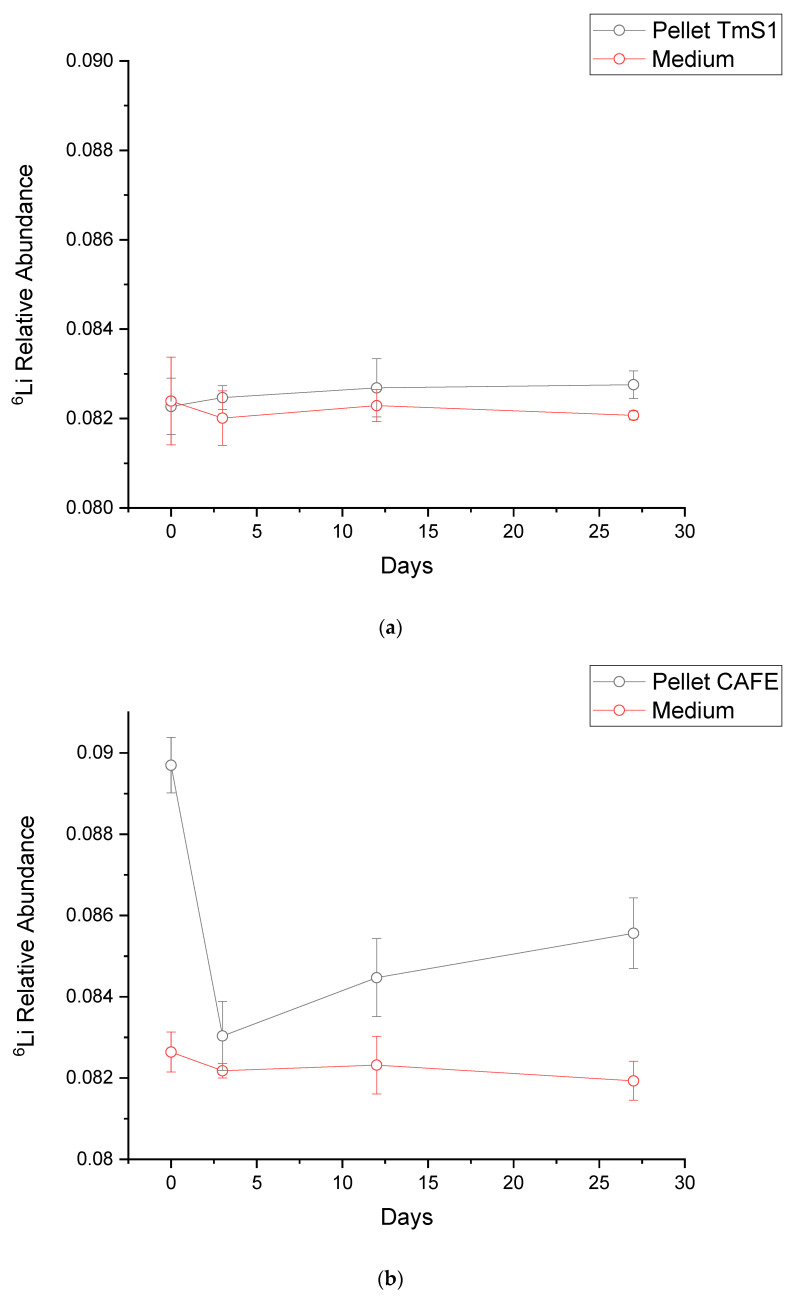

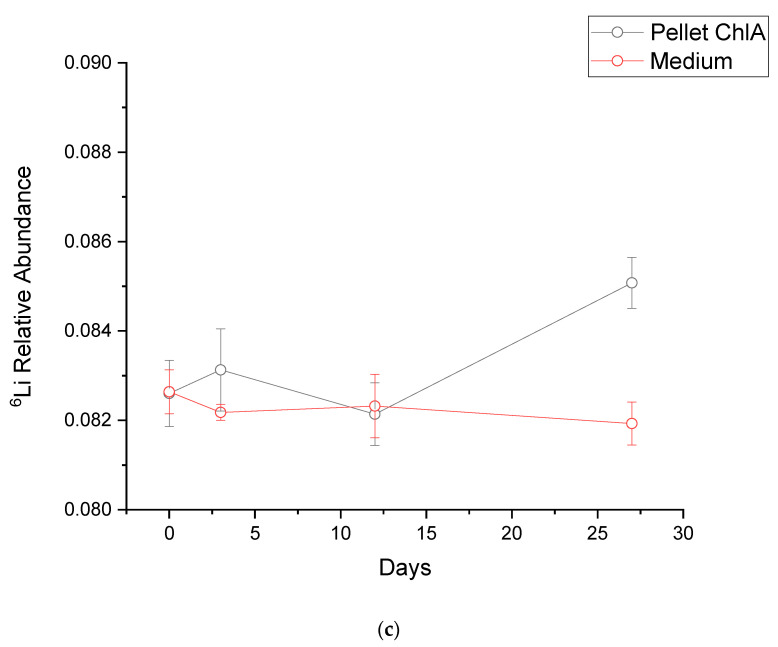
Isotopic relations for ^6^Li analysed in the trials with strains (**a**) TmS1, (**b**) CAFE, and (**c**) ChlA, and the observed fractionation on days 0, 3, 12, and 27, respectively. Black circles correspond to the pellet’s isotopic relation, while red circles correspond to the media.

Our three strains showed diverse behaviours in Li fractionation, with a preference for ^6^Li, as shown in Figure 2a–c. As stated by Balter and Vigier (2014) [22], ^6^Li has a more relevant role in biological systems than ^7^Li due to the greater diffusion rate of ^6^Li resulting from the difference in relative mass. Thus, a kinetic isotope effect during Li uptake by our microalgae could explain their preference towards the lighter Li isotope. However, our study does not delve deep into the reasons for Li fractionation, so we cannot ascertain that the kinetic isotope effect is the only process taking place. Opposite results should not be considered abnormal, as algae have previously shown a preference for ^41^K [72]—the heavier stable K isotope—with K being an element biologically related to Li. Additionally, studies in the different animal cells in mammals evidenced that Li isotopic fractionation varies depending on which organ each cell type belongs to [22]. Some species-specific characteristics are required to fully explain the different behaviours that have been observed, such as the composition of the cell wall, which is species-dependent [73], or the presence of transporters with isotopic differentiation capacity both for influx and efflux from cells. More research is required in this field to understand the reasons hidden behind this phenomenon and to expand the knowledge of each cell physiology and thus to predict possible fractionation outcomes. 

### 4.3. Biotechnological Perspectives

The experimental species were not selected, nor were they in previous contact with high Li concentrations. The two freshwater strains, ChlA and CAFE, were isolated from pristine environments where only trace Li levels are expected. TmS1 was isolated from the Mediterranean Sea, where Li concentrations around 200 µg/L are found [74], which are well below our experimental levels. Despite this, Li was captured and isotopically fractionated.

An artificial selection process could be conducted to obtain more efficient Li-capturing strains, which, in turn, could amplify—or not—the δ^6^. For instance, microalgae have been previously selected with the purpose of improving uranium uptake [64]. In our case, an artificial selection towards the obtainment of microalgae strains capable of greater Li capture could be achieved by selecting high-salinity resistant species that avoid the toxic effects of the ions by accumulating them in the vacuoles. However, artificial selection towards the obtainment of high-salinity resistant microalgae cells will not always correlate with an increase in ion capture, as some of the mechanisms are directed towards the ion efflux [68]. Another way to obtain microalgae with such properties could be through bioprospection in environments with high Li concentrations, such as in the Lithium Triangle. In this hypersaline region between Bolivia, Chile, and Argentina, some microalgae species have been detected [75,76]. Recently, a microalga isolated from the Atacama Desert (Chile), part of the Lithium Triangle, has been proposed as a tool for mining waste bioremediation [77]. Extremophile and extremotolerant species living there may possess mechanisms that could lead to increased capture or fractionation. Nevertheless, it must be stated that an almost perfect capture would not be desirable for fractionation purposes as the δ^6^ would be close to 0‰.

The fractionation outcomes, as long as the enrichment factor is high enough, would always be beneficial for biotechnological enrichment purposes, whether the microorganisms favour the lighter or heavier isotope. Given that the aim of the present research was to obtain ^6^Li, our three species provided this possibility. ^6^Li is concentrated in the cellular pellet, so with the removal of the microalgae, we would obtain ^6^Li-enriched lithium. With our CAFE strain, a δ^6^ of 85.4‰ was obtained moments after inoculation, which also had the maximum relative Li capture (8.76 µg per g of biomass), so this strain biomass could prove to be suitable as an enrichment tool. ChlA also showed a high δ^6^ of 38.4‰ at day 27, with an even greater Li relative capture than CAFE (31.66 µg per g of biomass). Complementarily, the liquid media would be enriched in ^7^Li. On the contrary, if the species took up ^7^Li preferentially, it would be the liquid matrix the one enriched in ^6^Li, whereas the pellet would contain concentrated ^7^Li.

For industrial up-scale, more species and times as well as other parameters such as light intensity, Li concentration, or culture media composition could be screened to reach optimal capture and fractionation.

## 5. Conclusions

Microalgae have the potential to become useful for Li enrichment. Though no great capture was achieved during this assay, the vast number of microalgae species and ample environmental conditions for the biotechnological process ensure that an optimal capture will eventually be detected. Additionally, if the Li pathways in microalgae are more widely studied, other approaches such as genetic engineering may be used for the obtainment of high-capturing high-fractionating microalgae strains. Nevertheless, as the Li capture approaches 100%, logically, the Li enrichment nears δ^6^ = 0‰, so a complete Li removal would not be desired for fractionation purposes.

In our assay, the maximum δ^6^ was observed with CAFE, a randomly chosen strain, thus pointing to the importance of extensive trials to detect biotechnologically valuable species.

In conclusion, even if knowledge is still limited, our results demonstrate that microalgae are capable of capturing Li and, in doing so, fractionate Li isotopes. Advances in this field might lead the way towards future nuclear biotechnology that will be able to fuel a future mix of electric generation with reliable nuclear (fission or/and fusion) reactors and variable renewables as a perfect combination of CO^2^ free emissions sources combined with advanced batteries for electric transportation that are urgently needed to fight climate change. The development of these technologies will require the massive use of Li isotopes, and Gaia could give us the cleanest and more sustainable tool for this fractionation.

## Figures and Tables

**Table 1:** Target genes and primers used for the 18S [62] and ITS [63] regions.

Gene	Primer Name	Primer Sequence
18S	18SF1	GGT TGA TYC TGC CAG TAG
	18SR1	GMW ACC TTG TTA CGA CTT
ITS	ITSu1	GGA AGK ARA AGT CGT AAC AAG G
	ITSu4	RGT TTC TTT TCC TCC GCT TA

**Table 2:** Li-mass quantification in each experimental pellet.

Strain	Day	Pellet Mass (g)	Li Mass in the Pellet (ng)	Li Mass/Biomass (µg/g)
TmS1	0	0.1273	887	6.96
TmS1	3	0.2861	1946	6.8
TmS1	12	0.1542	1113	7.21
TmS1	27	0.4174	3447	8.26
CAFE	0	0.0971	850	8.76
CAFE	3	0.1888	1265	6.7
CAFE	12	0.2261	1512	6.69
CAFE	27	0.5786	4597	7.94
ChlA	0	0.1214	885	7.29
ChlA	3	0.1432	784	5.48
ChlA	12	0.3876	1641	4.23
ChlA	27	0.1926	6098	31.66

**Table 3:** ^6^Li relative abundance and enrichment factor (δ^6^) in the experimental cultures.

		^6^Li/^7^Li Relative Abundance		
Strain	Time (Days)	Pellet (±Uncertainty)	Medium (±Uncertainty)	δ^6^
TmS1	0	0.08227 ± 0.00063	0.08239 ± 0.00098	−1.45
	3	0.08247 ± 0.00027	0.08201 ± 0.00061	5.58
	12	0.08269 ± 0.00065	0.08229 ± 0.00036	4.80
	27	0.08276 ± 0.00031	0.08207 ± 0.00010	8.35
CAFE	0	0.0897 ± 0.00068	008264 ± 0.00049	85.39
	3	0.08303 ± 0.00085	0.08218 ± 0.00018	10.39
	12	0.08447 ± 0.00096	0.08232 ± 0.00071	26.12
	27	0.08556 ± 0.00087	0.08193 ± 0.00048	44.32
ChlA	0	0.0826 ± 0.00074	0.08264 ± 0.00049	−0.44
	3	0.08313 ± 0.00092	0.08218 ± 0.00018	11.54
	12	0.08214 ± 0.00070	0.08232 ± 0.00071	−2.19
	27	0.08508 ± 0.00057	0.08193 ± 0.00048	38.40
